# Finerenone in Heart Failure—A Novel Therapeutic Approach

**DOI:** 10.3390/ijms252413711

**Published:** 2024-12-22

**Authors:** Amalie Holst-Hansen, Daniela Grimm, Markus Wehland

**Affiliations:** 1 Department of Biomedicine, Aarhus University, 8000 Aarhus, Denmark; 202104334@post.au.dk (A.H.-H.); dgg@biomed.au.dk (D.G.); 2 Department of Microgravity and Translational Regenerative Medicine, Otto von Guericke University, 39106 Magdeburg, Germany

**Keywords:** finerenone, heart failure, mineralocorticoid receptor antagonist, inflammation

## Abstract

This review will discuss heart failure, introduce a new drug finerenone, and discuss clinical studies with a focus on its effects on heart failure. Heart failure is a condition or syndrome characterized by an impairment of the pumping ability of the heart, thus no longer keeping up with the demands of the body. There are several types of heart failure; among them are heart failure with reduced ejection fraction, with mildly reduced ejection fraction and with preserved ejection fraction. Heart failure can be caused by several factors including lifestyle factors and diseases such as hypertension, type 2 diabetes mellitus and other cardiovascular diseases. Chronic kidney disease is also a risk factor of heart failure, as it leads to a state of inflammation that can impair the cardiovascular system over time. The novel nonsteroidal mineralocorticoid receptor antagonist finerenone antagonizes the mineralocorticoid receptor and thereby decreases the amount of fibrosis and inflammation that is observed in many heart failure patients. It shows an equal tissue distribution among heart and kidney, a high affinity and selectivity for the mineralocorticoid receptor and little risk of hyperkalemia and feminization. It also exhibits a reduction in the incidence of cardiovascular outcomes among patients with chronic kidney disease and type 2 diabetes mellitus. Therefore, finerenone has been proposed as a beneficial medication for reducing heart failure, especially in patients with diabetes and chronic kidney disease. Further studies are to be conducted to clarify the effects of finerenone alone and in combination with other drugs.

## 1. Introduction

In their 2021 guidelines, the Task Force for the Diagnosis and Treatment of Acute and Chronic Heart Failure of the European Society of Cardiology defines heart failure (HF) as “not a single pathological diagnosis, but a clinical syndrome consisting of cardinal symptoms (e.g., breathlessness, ankle swelling, and fatigue) that may be accompanied by signs (e.g., elevated jugular venous pressure, pulmonary crackles, and peripheral oedema). It is due to a structural and/or functional abnormality of the heart that results in elevated intracardiac pressures and/or inadequate cardiac output at rest and/or during exercise” [1]. There are different types of this syndrome including HF with reduced ejection fraction (HFrEF), HF with mildly reduced ejection fraction (HFmrEF) and HF with preserved ejection fraction (HFpEF). Furthermore, HF is also divided into different functional classes according to the New York Heart Association (NYHA) [2].

There are several common risk factors for HF such as hypertension, diabetes mellitus and smoking, among others. In addition, a high degree of inflammation, which is often seen in kidney disease patients, has been shown to be a risk factor for HF. The drug finerenone aims to reduce this risk factor, especially in patients with chronic kidney disease (CKD) and diabetes [3].

Finerenone is a selective mineralocorticoid receptor antagonist (MRA). Because of its high selectivity, the drug has shown fewer side effects like feminization and hyperkalemia than other MRAs, such as spironolactone [4].

Finerenone inhibits the effects of mineralocorticoids such as aldosterone. Aldosterone has several effects on the body. In the kidney, it leads to more sodium reabsorption and thus more water retention and a higher blood pressure (BP) [4]. Aldosterone, and therefore also finerenone, show effects on the heart and vasculature. It has been demonstrated that aldosterone increases the degree of inflammation and fibrosis in the heart by stimulating several different cell types. The application of finerenone may reduce the degree of cardiac inflammation and fibrosis and thus improve cardiovascular outcomes (CVO) and decrease the risk of HF [5].

The objective of this review is to describe the new drug finerenone and to present and discuss clinical trials investigating its effects on CVO, especially on HF.

## 2. Heart Failure

### 2.1. Classes of Heart Failure

HF is defined as a condition where the heart is not capable of pumping the adequate amount of blood that is needed for the body to function. Generally seen, HF is divided into left-sided HF and right-sided HF [6].

There are more types of left-sided HF, based on whether the ejection fraction (EF) is affected or not. The left ventricular ejection fraction is the quotient of the left ventricular end-diastolic volume and the left ventricular end-systolic volume, which can be obtained from analyzing apical four- and two-chamber views during transthoracic echocardiography [7]. HFrEF, also known as systolic HF, is defined as HF where the EF is equal to or lower than 40% (the normal ejection fraction is 55–70%). This type of HF occurs when the left ventricle loses its ability to contract sufficiently. HFpEF, also known as diastolic HF, is defined as HF where the EF is equal to or higher than 50%. This type of HF occurs when the left ventricle loses its ability to relax sufficiently to fill up with an adequate amount of blood between each heartbeat. There is also a middle group called HF with mildly reduced ejection fraction, also known as HF with mildly reduced ejection fraction (HFmrEF), where the EF is between 41% and 49%. Furthermore, if the patient exhibits an EF under 40% and a follow-up measurement shows an EF above 40% this can be classified as HF with improved ejection fraction (HFimpEF) [2,6].

Right-sided HF can be a consequence of left-sided HF. Left-sided HF leads to the accumulation of blood in the arteries of the lungs resulting in a higher BP in those arteries, which damages the right side of the heart [6]. However, an isolated right ventricular failure without impaired left ventricular function can also occur, caused by lung diseases such as a massive acute pulmonary embolism with more than 50% thrombotic occlusion of pulmonary vasculature or chronic respiratory disorders such as COPD, both increasing right ventricular afterload [8,9].

HF can be divided into different stages, which have been defined in collaboration between the American Heart Association (AHA) and the American College of Cardiology:

1. Stage A (at risk for HF): No symptoms or structural or functional heart diseases, but risk factors such as hypertension, diabetes or family history of cardiomyopathy.
2. Stage B (pre-HF): No symptoms, but either structural heart disease (e.g., ventricular hypertrophy), increased filling pressure or other risk factors.
3. Stage C (symptomatic HF): Symptoms, either current or previous, as well as structural heart disease.
4. Stage D (advanced HF): HF symptoms that affect daily life and/or lead to several hospitalizations [2,10].

Stages C or D can further be divided into one of the four NYHA classes, which are listed in Table 1 [10,11].

### 2.2. Risks of HF

Anyone can develop HF, but the risk increases with age. Several lifestyle factors also contribute to an increased risk of HF including smoking, obesity and alcohol or drug abuse. Furthermore, other diseases such as type 2 diabetes mellitus (T2DM) and especially other cardiovascular diseases, for instance, coronary artery disease and hypertension, are associated with an increased risk of HF [12,13]. In addition, conditions that damage the heart or force the heart to work harder lead to a higher risk of HF. These conditions include previous myocardial infarcts and heart muscle diseases due to for example viral infections [14]. Furthermore, patients with CKD also exhibit an increased risk of HF. CKD causes a systemic state of proinflammation that results in constant vascular and myocardial remodeling and thereby increased myocardial fibrosis and calcification of arteries and valves that induce weakening of the cardiovascular system. It was found that CKD patients have a higher risk of cardiovascular mortality due to, amongst other things, stroke or myocardial infarction, especially in advanced CKD stages of HF [3].

### 2.3. Symptoms of HF

Because HF is a complex clinical diagnosis, its symptoms can be very broad. There are symptoms arising from a dysfunction in blood ejection or ventricular filling. One symptom of HF is edema due to blood accumulating in the veins, leading to plasma being filtrated to the tissue. Blood accumulating in the pulmonary veins leads to symptoms of HF as well. Due to plasma being pushed out in the alveoli of the lungs, the exchange of oxygen and CO_2_ is inhibited, and the patient can experience shortness of breath as well as wheezing and coughing. The fact that the heart can no longer pump enough blood to all the parts of the body leads to vasoconstriction in large parts of the body to keep up the BP and support blood flow to essential organs such as brain and heart. This is caused by an increased sympathetic nervous system activity, which also explains why the heart beats faster. Therefore, the stomach receives less blood, which can cause nausea and weight loss. The limbs also receive less blood, which contributes to a feeling of fatigue [2,15].

### 2.4. Diagnosis of HF

Several tests contribute to the diagnosis of HF. A physical examination including stethoscoping for heart murmur, BP measurement, electrocardiography as well as examination of symptoms such as edema, and receiving a thorough patient history is the first step. Furthermore, imaging tests such as echocardiography and computed tomography scan as well as blood samples testing for levels of B-type natriuretic peptide (BNP) in combination with an exercise stress test can assist in determining the type, stage and class of the patient’s HF [7,16]. It can be challenging to diagnose HFpEF and therefore the European Society of Cardiology has proposed a four-step diagnostic algorithm with both ambulatory measurements and investigations performed by a cardiologist [17].

### 2.5. Treatment of HF

Several treatment options for HF depending on its type, comorbidities and other conditions of the patient are known. Besides support and education about HF, as well as exercise and a healthy diet, there are several medical treatment options. Angiotensin-converting enzyme inhibitors (ACE inhibitors), angiotensin receptor blockers (ARBs), angiotensin receptor-neprilysin inhibitors (ARNIs), beta-adrenoceptor antagonists (BBs), sodium-glucose co-transporter 2 inhibitors (SGLT2-is), mineralocorticoid receptor antagonists (MRAs) and diuretics are the seven drug classes that are used in HF patients [18].

### 2.6. Standard Medical Treatment Recommendations

The recommended medical treatment regimen depends on the type of HF. A combination of ARNI, BBs, SGLT2-is and MRAs is recommended for patients with HFrEF. If an ARNI is too expensive or cannot be tolerated by a patient, it can be switched to either ACE inhibitors or ARBs. Treatment with devices should be considered in each patient individually. An implantable cardiac defibrillator could be recommended in patients with an EF under 35% [18]. Patients with both CKD and HF were typically excluded from trials examining HF. However, analyses of subgroups show that the application of ACE inhibitors in combination with BBs results in a reduction in mortality in these patients. If treatment with these two drug types is insufficient, MRAs can be considered. This should be carried out with care in patients with advanced kidney disease because of the risk of hyperkalemia. If the patient still has symptoms with a combination of these three drug types, additionally neprilysin inhibitors (ARNIs) are recommended [3]. In HFmrEF patients, the first line therapy is SGLT2-is, while the second line therapy is any combination with either ARNIs, ARBs or ACE inhibitors as well as MRAs and BBs. HFpEF patients are a challenging and complex group of patients and the recommended treatment for these patients is to treat the comorbidities of their HF. For example, treating a patient with diabetes and HFpEF with a SGLT2-i. HFimpEF patients should still receive medical therapy because due to the fact that the mechanisms leading to HF are still present in the patient, they are at risk of relapse [18].

## 3. Finerenone

### 3.1. Structure

**Figure 1:**
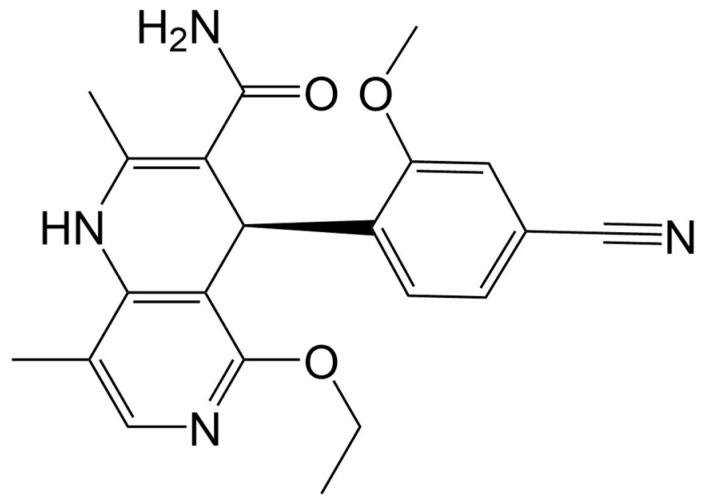
Structural formula of finerenone.

Finerenone is a nonsteroidal MRA with the molecular formula C_21_H_22_N_4_O_3_ [19]. The structural formula of finerenone is shown in Figure 1.

### 3.2. Mechanism of Action

**Figure 2:**
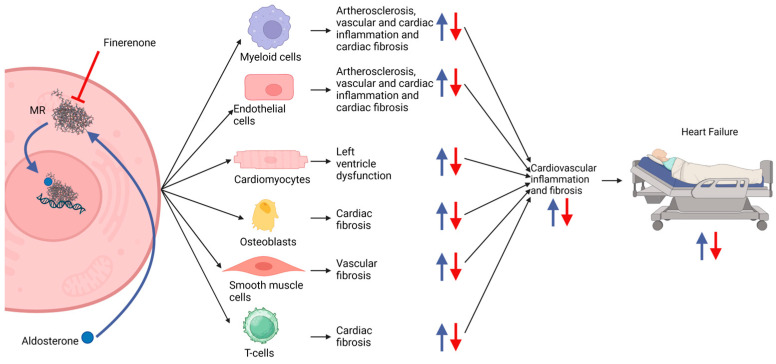
Proposed mechanisms of increased degree of inflammation and fibrosis in HF patients. Myeloid cells, endothelial cells, cardiomyocytes, osteoblasts, smooth muscle cells and T-cells activated by aldosterone through their MRs contribute to an increasing inflammation and fibrosis in the cardiovascular system that can lead to HF (blue arrows). Finerenone antagonizes the MR and thus decreases the degree of inflammation and fibrosis (red arrows) [20,21,22]. The figure was made with https://www.biorender.com (accessed on 19 December 2024).

Finerenone acts on the MRs, which are abundantly present in the heart, vasculature, and the principal cells of the kidney [5]. It is proposed that the main benefit of finerenone is driven by a reduction in inflammation and fibrosis in the heart and kidney. This reduction happens because of the inhibition of downstream mechanisms initiated by the aldosterone-mediated activation of the MR. MRs in several different cell types such as cardiomyocytes and myeloid cells are involved in inflammation and fibrosis in the heart and vascular system leading to HF [20,21,22]. An overview of the mechanisms of aldosterone in inducing HF is illustrated in Figure 2.

### 3.3. Trials

Until now, there have been published three phase 3 trials investigating finerenone: “Finerenone in Reducing Kidney Failure and Disease Progression in Diabetic Kidney Disease” trial (FIDELIO-DKD) (NCT02540993) with 5734 participants, “Finerenone in Reducing Cardiovascular Mortality and Morbidity in Diabetic Kidney Disease” (FIGARO-DKD) (NCT02545049) with 7437 participants, and “Study to Evaluate the Efficacy (Effect on Disease) and Safety of Finerenone on Morbidity (Events Indicating Disease Worsening) & Mortality (Death Rate) in Participants With Heart Failure and Left Ventricular Ejection Fraction (Proportion of Blood Expelled Per Heart Stroke) Greater or Equal to 40%” (FINEARTS-HF) (NCT04435626) with 6016 participants. They compared finerenone to a placebo on top of a renin–angiotensin system blockade or standard therapy, respectively. FIGARO-DKD had a primary outcome measure as a composite of death from cardiovascular causes, nonfatal myocardial infarction, nonfatal stroke or hospitalization for HF. Between the two groups, there was a hazard ratio (HR) of 0.87 (95% CI of 0.76 to 0.98; *p* = 0.03) in the primary outcome. FIDELIO-DKD had the same outcomes as a key secondary outcome and between the two groups, there was an HR of 0.86 (95% CI; 0.75–0.99; *p* = 0.03) in this outcome. The two trials also showed a modest effect of finerenone in reducing BP. The effects on the CVO appeared earlier than the effects on the kidney outcomes (seen in the separation of Kaplan–Meier curves at 4 and 12 months, respectively, in FIDELIO-DKD). The primary outcome of FINEARTS-HF was a composite endpoint of the number of cardiovascular deaths and heart failure events, with secondary outcome measures such as time to total (first and recurrent) HF events, improvement in NYHA class from baseline to month 12, change in total symptom score, time to first occurrence of composite renal endpoint, and time to death from any cause. This trial also could show significantly lower rates of primary outcome events in the finerenone group compared to the placebo group (rate ratio 0.84; 95% CI, 0.74 to 0.95; *p* = 0.007) with stronger effects on the number of worsening heart failure events (rate ratio 0.82; 95% CI 0.71 to 0.94; *p* = 0.006) than on cardiovascular death (hazard ratio 0.93; 95% CI 0.78 to 1.11) [23,24,25,26]. Efficacies did not differ between men and women [27].

This data suggests that to some extent the benefit of finerenone is also due to hemodynamic mechanisms of action driven by for instance reduction in vascular stiffness as well as the diuretic effects [5,20,28,29,30,31,32,33]. Even though finerenone has a short half-life of approximately 2 h, the “Mineralocorticoid Receptor Antagonist Tolerability Study—Diabetic Nephropathy” (ARTS-DN) (NCT01874431), which studied placebo-adjusted changes in 24 h ambulatory SBP among 240 patients, showed that once-daily-administered finerenone results in a steady BP decrease through all 24 h of the day [34]. However, the hemodynamic effects are probably of a minor character and the main effects are expected to be driven by the inhibition of inflammation and fibrosis in heart and kidney [5,20,28,29,30,31,32,33].

### 3.4. Indications and Contraindications for the Use of Finerenone

Spironolactone and eplerenone (steroidal MRAs) are included in the recommendations for patients with HFrEF. However, these two drugs have the side effect induction of hyperkalemia, which particularly is a high risk for patients with CKD and diabetes mellitus. Due to the risk of hyperkalemia, spironolactone and eplerenone are often not used in this group of patients. Nevertheless, studies showed that the benefits from these drugs in terms of reduction in CVO are still present in this patient group.

The mineralocorticoid Receptor Antagonist Tolerability Study (ARTS) (NCT01345656), a phase 2 trial, found that compared to a dose of 25–50 mg/day spironolactone, a dose of finerenone of 10 mg once daily or 5 mg twice daily leads to the same level of decrease in BNP and amino-terminal proBNP (indications of HF). However, compared to spironolactone, finerenone had a lower effect on serum potassium levels. Both fewer incidences of hyperkalemia as well as a lower mean potassium concentration were observed in the finerenone patients than in the spironolactone patients. Therefore, it could be a good therapy option for the patient group with HF, CKD and T2DM [35,36,37,38].

A pooled analysis of the FIGARO-DKD and FIDELIO-DKD trials named finerenone in chronic kidney disease and type 2 diabetes: combined FIDELIO-DKD and FIGARO-DKD Trial Programme Analysis (FIDELITY) investigated the efficacy of finerenone among subgroups. The trial found no difference in the effect of finerenone in subgroups such as eGFR-categories, differences in baseline HbA1c or baseline insulin use as well as diabetes duration [39,40,41].

The European Medicines Agency (EMA) stated that contraindications for the use of finerenone are hypersensitivity to the drug, Addison’s disease and the simultaneous use of strong CYP3A4-inhibitors because finerenone is mainly cleared by CYP3A4. Ingestion of grapefruit and its juice should also be avoided due to its inhibitory effect on CYP3A4. Furthermore, it is not recommended to use finerenone in combination with other medical products that increase serum potassium [42].

### 3.5. Safety and Adverse Effects

No statistically significant differences in the incidence of treatment-emergent adverse effects (TEAE) between the groups of patients receiving finerenone and placebo were found in the FIDELIO-DKD and FIGARO-DKD studies. However, in FIDELIO-DKD, the incidence of hyperkalemia-related adverse events was twice as high in the finerenone group as in the placebo group (18.3% vs. 9%), but there were no fatal hyperkalemia events in any of the groups. The FIGARO-DKD also showed a doubling in the incidence of hyperkalemia in the finerenone group in comparison to the placebo group with an incidence of hyperkalemia at 10.8% in the finerenone group vs. 5.3% in the placebo group. But the incidence of discontinuation (1.2% vs. 0.4%) and hospitalization (0.6% vs. 0.1%) was low in both groups [29,32]. It is not seldom that medication for HF patients can cause hyperkalemia. In other studies, investigating the effects of RAS inhibition with renin inhibitors, ARBs and ACE inhibitors, there were higher percentages of patients being removed from the trial due to hyperkalemia than in FIDELIO-DKD [29]. Furthermore, as mentioned earlier, the results in ARTS show that finerenone exhibits lower effects on serum potassium levels than spironolactone [37].

In the other phase 2b trial, MinerAlocorticoid Receptor antagonist Tolerability Study-Heart Failure ARTS-HF (NCT01807221), comparing different doses of finerenone to eplerenone, there were no statistically significant differences in the incidence of TEAE between the finerenone groups and the eplerenone group nor a significant difference in the incidence of hyperkalemia. The finerenone group had a reduction of 30% or more in proBNP from baseline to day 90 in a similar proportion of the participants as in the eplerenone group [35,43]. FINEARTS HF reported an increased risk of hyperkalemia and a reduced risk of hypokalemia in patients receiving finerenone compared to placebo [23]. The incidence of gynecomastia in FIGARO-DKD was low (0.1% in both finerenone and placebo group), which is a side effect seen from other MRAs such as spironolactone [18,32]. The safety profile and tolerability were similar in both male and female participants [27]. An overview of studies included in this review based on the systematical search is given in Table 2.

## 4. Discussion

Both the FIDELIO-DKD and FIGARO-DKD trials showed that finerenone on top of a renin–angiotensin system blockade or standard therapy has an effect in the reduction in CVO compared to placebo on top of a renin–angiotensin system blockade [5,29,32]. The primary outcome of HR in FIGARO-DKD was driven mainly by a reduction in hospitalization for HF. This indicates that among patients with CKD and T2DM, finerenone has positive effects on HF [32]. This is supported by the reduction in the key secondary composite outcome seen in the FIDELIO-DKD [29]. A benefit of finerenone compared to other MRAs is its high selectivity for the MR. Finerenone binds 500-fold more selectively to MRs than to other steroid hormone receptors. In comparison, spironolactone only binds 3 times more to the MR than to the androgen receptor [34]. This selectivity can cause fewer side effects such as gynecomastia, which is a common side effect of spironolactone [18]. In the FIGARO-DKD, few incidences of gynecomastia were seen in both the finerenone and placebo groups, suggesting that this assumption is correct [32]. Finerenone also showed a better affinity for the MR than the other MRA eplerenone [35]. A side effect of finerenone seen in FIGARO-DKD, FIDELIO-DKD and FINEARTS-HF is hyperkalemia. But the incidences of hyperkalemia-related discontinuation in the finerenone groups were quite low in FIDELIO-DKD (2.3%) and in FIGARO-DKD (1.2%) [29,32]. In the ARTS study, where finerenone was compared to spironolactone, lower incidences of hyperkalemia and lower mean serum potassium concentrations were observed in the finerenone group compared to the spironolactone group [37]. This difference was not seen in ARTS-HF, comparing finerenone and eplerenone. This result suggests that finerenone can be more beneficial than spironolactone in patients with an increased risk of hyperkalemia such as patients with T2DM and CKD [35]. Furthermore, finerenone showed an equal tissue distribution between kidney and heart, unlike the steroidal MRAs spironolactone and eplerenone, which both accumulate in the kidney. This suggests that finerenone revealed more beneficial effects in HF outcomes than the steroidal MRAs [44]. Furthermore, an analysis of new-onset atrial fibrillation or flutter (AFF) in the FIDELIO-DKD trial suggested a reduction in these cases by finerenone compared to placebo (hazard ratio: 0.71; 95% confidence interval: 0.53–0.94; *p* = 0.016), irrespective of the patient’s AFF history [45]. However, a recent large meta-analysis of 23 MRA trials revealed that MRAs, in general, reduced the incidence of new-onset AFF significantly compared to placebo (hazard ratio:0.75; 95% confidence interval: 0.66–0.87; *p* = 0.001), but this effect was exclusively driven by spironolactone, while eplerenone and finerenone showed only neutral effects. HF status did not influence the findings [46].

Taken together, the high degree of selectivity and affinity, the relatively low risk of side effects as well as the equal tissue distribution of finerenone suggest that finerenone is a viable alternative to the steroidal MRAs. So far, finerenone is approved for the treatment of HF in adults with both CKD and T2DM in the United States, with a decision of EMA still pending [34].

The use of SGLT2-is is currently under research for beneficial effects in HF patients and the subgroup using SGLT2-is and receiving finerenone in FIGARO-DKD demonstrated greater beneficial effects on the composite outcome of cardiovascular death and hospitalization for HF. Unfortunately, the subgroup was very small, and this should be further examined before anything is concluded [5]. There is a study planned to examine the effect of SGLT2-is and finerenone in combination (CONFIRMATION-HF) (CONFIRMATION) (NCT06024746) [47]. Two more studies are planned to study the effect of finerenone in combination with other drugs. (PolyPreventHF) (NCT06143566) will investigate the effect of a combination of empagliflozin, losartan and finerenone in patients with T2DM and a high risk of HF [48], while (Steno1) (NCT06082063) will investigate the effect of a multifactorial intervention with among others semaglutide, sotagliflozin, finerenone, ezetimibe and/or PCSK9-inhibitors in patients with type 1 diabetes at high risk of cardiovascular diseases [49]. These and further studies will clarify the effects of finerenone alone and in combination with other treatment options.

It should also be mentioned that the completed studies included in this review all have been funded by Bayer (the pharmaceutical company behind finerenone). More studies independent of Bayer should be conducted to ensure the accuracy of the results. As of now (PolyPreventHF), (Steno1) and an observational study (NCT05974566) are the only studies on clinicaltrials.gov, which does not state a collaboration with Bayer [48,49,50]. An overview of trials registered on clinicaltrials.gov can be seen in Table 3.

Finally, there are indications from several animal studies that finerenone has antioxidant abilities. It has been shown that finerenone decreased myocardial ROS production and increased NO bioavailability in Zucker fa/fa rats, resulting in both cardiac and renal function improvements [51]. In addition, aortic ring protein expression of Mn-SOD and Cu/Zn-SOD as well as renal total SOD activity was increased in finerenone-treated Munich Wistar Frömter rats [52]. In a myocardial infarction mouse model, finerenone improved left ventricular compliance and elasticity as well as endothelial function and reduced interstitial fibrosis. Moreover, low-dose angiotensin-II-induced oxidative stress was attenuated by finerenone in isolated coronary arteries from non-infarcted mice. It is likely that these effects also contribute to the observed beneficial cardiorenal effects of finerenone in clinical trials; however, no detailed analyses have been conducted regarding HF so far.

Due to the designs of the discussed studies and the fact that finerenone is a relatively new drug that has only recently been considered for HF treatment, there remains the question of long-term HF-related benefits and side effects under continued finerenone administration. So far, no follow-up data exceeding a timespan of just a few years are available; therefore, all considerations can only be based on extrapolations, which must be interpreted with caution. While no further investigations have been performed assessing possible long-term side effects, Vaduganathan et al. [53] processed data from the FINEARTS-HF trial in a prespecified analysis using validated nonparametric actuarial methods such as age-based Kaplan–Meier curves using age at randomization rather than time from randomization, to estimate survival times free from primary endpoint events. Mean event-free survival for a 55-year-old patient was estimated to be 13.6 years (95% CI, 11.9–15.2 years) under finerenone therapy compared to placebo with only 10.5 years (95% CI, 6.8–11.3 years), resulting in an increase by 3.1 years (95% CI, 0.8–5.4 years; *p* = 0 .007). For a 65-year-old patient, they calculated a gain of 2.0 years (95% CI, 0.8–3.3 years; *p* = 0.001). This effect was also observed in patients who received an SGLT2-i prior to finerenone (65-year-old patient: 3.1 years; 95% CI, 0.1–6.0 years; *p* = 0.04) [53]. However, long-term follow-up studies have to be performed to test whether these theoretical findings are clinically relevant.

Lastly, it remains to be seen whether finerenone might extend its usefulness in HF treatment and prevention to patient groups not suffering from diabetes or CKD. So far, no trials have been conducted to specifically address this question, however, Pamporis et al. noticed in their network meta-analysis of 32 randomized MRA trials, that almost half of the population of the finerenone trials did not have T2DM and that this might be an indicator for a clinical benefit that transcends this subgroup [54].

Overall, the currently available data seem to justify introducing finerenone into HF therapy and amending the different guidelines accordingly. The biggest potential of the drug seems to be the management of HFmrEf and HFpEF and cases where steroidal MRAs could not be used [54]. However, it should be noted that for patients with CKD and T2DM, SGLT2-is are available, which outperform finerenone in the reduction in renal outcomes and HF hospitalizations [55]. It is therefore possible that finerenone will find its niche as a combination drug, as is currently being tested in the CONFIRMATION trial.

## 5. Methods

**Figure 3:**
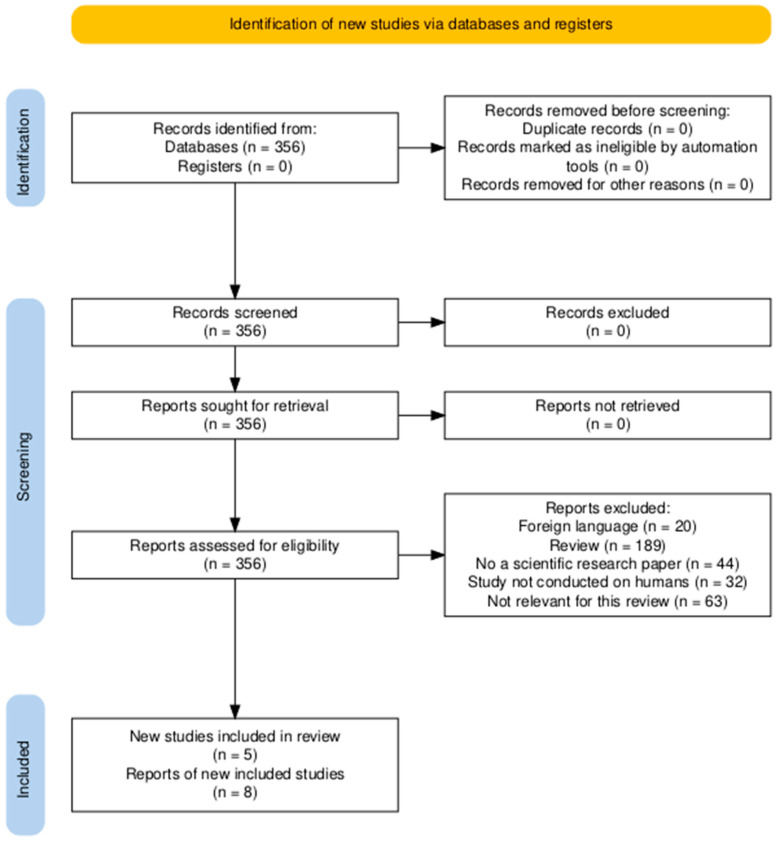
PRISMA flow diagram of the primary literature in this review. Made from https://www.prisma-statement.org/prisma-2020-flow-diagram (accessed on 19 December 2024) [56].

The literature used in this review was found through searches on PubMed, Clinicaltrials.gov, and a few online pages. A systematic search on PubMed was made using the term: (((Finerenone) OR (kerendia)) OR (“BAY 94-8862”)) AND ((((((“heart failure”) OR (Cardiovascular)) OR (Cardiorenal)) OR (Hypertension)) OR (“Heart Failure”[Mesh])) OR (“Cardiovascular Diseases”[Mesh])). Another trade name of finerenone is “Firialta”, but this was not included in the search because it did not have any hits. The process of sorting the articles is illustrated in the PRISMA diagram below (Figure 3). Other literature from references in reviews and original papers has been included as well to ensure a broader amount of information on finerenone.

Furthermore, a search on clinicaltrials.gov with “Finerenone”, “Kerendia”, “BAY 94-8862” and “Firialta” as intervention/treatment and “Heart failure” as condition/disease has been undertaken.

## 6. Conclusions

The trials have shown that patients receiving finerenone on top of a renin–angiotensin system blockade or standard therapy have a lower incidence of CVO including HF than those receiving a placebo on top of a renin–angiotensin system blockade. Finerenone also seems to lead to less gynecomastia and hyperkalemia as compared with spironolactone. The low risk of side effects, equal tissue distribution, and high selectivity and affinity for the MR propose that finerenone is a promising MRA. The effect of finerenone is probably mainly driven by the inhibition of inflammatory and fibrotic mechanisms that normally are initiated when the MR is activated. A smaller part of the effect can also be attributed to the modest effect on BP as well as antioxidative properties. Further clinical and translational animal studies are necessary to clarify the effects of finerenone by itself and in combination with other drugs as well as to identify possible, so far unknown, mechanisms or factors that might confer beneficial pleiotropic effects.

## Figures and Tables

**Table 1:** Classes of symptomatic HF.

Class	Description
I: “No limitation of physical activity”	Comfortable at both rest and ordinary physical activity with no fatigue or shortness of breath.
II: “Slight limitation of physical activity”	Comfortable at rest, but ordinary physical activity causes fatigue and shortness of breath.
III: “Marked limitation of physical activity”	Comfortable at rest, but activities that are less demanding than ordinary physical activity cause fatigue and shortness of breath.
IV: “Unable to perform any physical activity without symptoms”	Any physical activity causes fatigue, shortness of breath and further discomfort.

Compiled from [10,11].

**Table 2:** Overview of concluded finerenone trials included in this review.

Name	Design	Results	Conclusions
Finerenone in Reducing Cardiovascular Mortality and Morbidity in Diabetic Kidney Disease FIGARO-DKD NCT02545049 [5,32,33]	Phase 3 trial, international randomized double-blinded controlled trial (48 countries) 7437 participants	Primary outcome showed a hazard ratio of 0.87 (95% CI of 0.76 to 0.98; *p* = 0.03). This positive HR (against finerenone) was mainly a result of the reduction in the incidence of hospitalization for HF. Difference between groups in mean SBP was 3.5 mmHg at month 4 and 2.6 mmHg at month 24. There were no significant differences in TEAE between the finerenone group and the placebo group, but there was a higher incidence of hyperkalemia among people in the placebo group. There were low incidences of gynecomastia in both the finerenone and placebo groups.	Finerenone showed a reduction in especially hospitalization for HF as well as a small reduction in SBP. The trial suggests that finerenone can cause hyperkalemia, but this led to few cases of discontinuation and hospitalization. Gynecomastia does not seem to be a side effect of finerenone.
Finerenone in Reducing Kidney Failure and Disease Progression in Diabetic Kidney Disease trial FIDELIO-DKD NCT02540993 [20,28,29,30,31]	Phase 3 trial, international randomized double-blinded controlled trial (48 countries) 5734 participants	The hazard ratio of the key secondary outcome between the two groups was HR = 0.86 (95 % CI; 0.75–0.99; *p* = 0.03). Finerenone also showed a small reduction in BP. There was no significant difference in the incidence of serious adverse events between the two groups. The incidence of hyperkalemia-related adverse events was twice as high in the finerenone group as in the placebo group (18.3% vs. 9%), but there were no fatal hyperkalemia events in any of the groups. No difference in the treatment effect or adverse effects among subgroups was found.	Finerenone showed an effect on combined cardiovascular outcomes. Due to early seen effects on these outcomes as well as the small reduction in BP, finerenone might have a mode of action through hemodynamic effects as well as its anti-inflammatory and antifibrotic effects. Finerenone can cause hyperkalemia, but other side effects cannot be correlated to finerenone in this trial.
The Mineralocorticoid Receptor Antagonist Tolerability Study-Diabetic Nephropathy ARTS-DN NCT01874431 [34]	Phase 2b trial, multicenter randomized controlled trial, double-blinded 823 participants, 240 were selected for a 24 h ambulatory BP measurement	Placebo-adjusted changes in 24-h ambulatory SBP at day 90 were −8.3 mmHg, −11.2 mmHg, and −9.9 mmHg for finerenone 10, 15, and 20 mg, respectively. Similar effects were seen on the daytime and nighttime ambulatory BP measurements, separately. There was no statistically significant difference in nocturnal dipping between the two groups.	This study shows that once-daily administrated finerenone results in a steady BP decrease through all 24 h a day. The sample size and the duration of the study, however, were small.
Mineralocorticoid Receptor Antagonist Tolerability Study-Heart Failure ARTS-HF NCT01807221 [27,35,43]	Phase 2b, randomized, double-blind multicenter study (25 countries) 1066 participants	Different doses of finerenone were compared to eplerenone. The finerenone groups revealed a similar decrease in proBNP as the eplerenone group. There was a small decrease in the SBP from baseline to day 90 in all groups. There was no statistically significant difference in the incidence of TEAE between the finerenone groups and the eplerenone group, nor a significant difference in the incidence of hyperkalemia.	Finerenone showed the same safety profile as eplerenone as well as a reduction in proBNP and cardiovascular outcomes.
The minerAlocorticoid Receptor Antagonist Tolerability Study ARTS NCT01345656 [37,38]	Randomized phase 2, multicenter double-blinded controlled trial that was divided into 2 parts (10 countries) Part A: Safety and tolerability test with 65 participants Part B: Effectiveness test (comparison to placebo and open-labeled spironolactone 25 or 50 mg daily) with 392 participants	Finerenone leads to the same level of decrease in BNP and amino-terminal proBNP as spironolactone. Patients receiving 5 mg finerenone twice daily or 10 mg finerenone once daily had a significantly higher mean increase in serum potassium levels than the placebo group. Compared to spironolactone finerenone exerted a lower effect on the serum potassium levels.	Finerenone showed the same effect on BNP and proBNP as spironolactone. The half-life of finerenone is quite short (approximately 2 h) but the trial showed no advantages in twice daily administrations in comparison to once daily administration. The study showed that finerenone can lead to hyperkalemia but suggested that this side effect is less common in finerenone than in spironolactone.
Study to Evaluate the Efficacy (Effect on Disease) and Safety of Finerenone on Morbidity (Events Indicating Disease Worsening) & Mortality (Death Rate) in Participants With Heart Failure and Left Ventricular Ejection Fraction (Proportion of Blood Expelled Per Heart Stroke) Greater or Equal to 40% FINEARTS-HF NCT04435626 [23,24,25,26]	Phase 3 multicenter, randomized, double-blind, parallel-group, placebo-controlled 20 mg or 40 mg finerenone once daily vs. placebo in addition to usual therapy 6016 participants	Overall, after 32 months, 1083 primary outcome events were recorded in 624 patients of the 3003 who received finerenone, while placebo treatment resulted in 1283 primary outcome events in 719 of the 2998 patients (rate ratio 0.84; 95% CI 0.74 to 0.95; *p* = 0.007). Worsening heart failure events were 842 vs. 1024 in the finerenone vs. placebo groups (rate ratio, 0.82; 95% CI 0.71 to 0.94; *p* = 0.006) and death rates 8.1% vs. 8.7%, respectively, hazard ratio 0.93; 95% CI 0.78 to 1.11). Finerenone increased the risk for hyperkalemia and reduced the risk for hypokalemia.	For patients with HFpEF or HFrEF treatment with finerenone significantly reduced a composite of total worsening heart failure events and death from cardiovascular causes.

**Table 3:** Overview of planned and ongoing trials.

Name	Design	Phase
A Study to Determine the Efficacy and Safety of Finerenone on Morbidity and Mortality Among Hospitalized Heart Failure Patients (REDEFINE-HF) NCT06008197	Randomized controlled trial 5200 participants to be enrolled (estimated)	recruiting
A Study to Determine the Efficacy and Safety of Finerenone and SGLT2i in Combination in Hospitalized Patients With Heart Failure (CONFIRMATION-HF) NCT06024746	Randomized controlled trial 1500 participants to be enrolled (estimated)	not yet recruiting
A Study to Evaluate Finerenone on Clinical Efficacy and Safety in Patients With Heart Failure Who Are Intolerant or Not Eligible for Treatment With Steroidal Mineralocorticoid Receptor Antagonists (FINALITY-HF) NCT06033950	Randomized controlled trial 2600 participants to be enrolled (estimated)	not yet recruiting
The EFfect of FinErenone in Kidney TransplantiOn Recipients: The EFFEKTOR Study NCT06059664	Randomized controlled trial 150 to be enrolled (estimated)	recruiting
Polypill for Prevention of Cardiomyopathy (PolyPreventHF) NCT06143566	Randomized pilot study 60 to be enrolled	recruiting
Multifactorial Intervention to Reduce Cardiovascular Disease in Type 1 Diabetes (Steno1) NCT06082063	PROBE design 2000 to be enrolled	not yet recruiting
Efficacy and Safety of Finerenone in Heart Failure With Reduced Ejection Fraction NCT05974566	Cohort study 60 to be enrolled	not yet recruiting

## Abbreviations

ACE	Angiotensin-converting enzyme
AHA	American Heart Association
AFF	Atrial fibrillation or flutter
ARB(s)	Angiotensin receptor blocker(s)
ARNI(s)	Angiotensin receptor—neprilysin inhibitor(s)
BB(s)	Beta adrenoceptor antagonists
BNP	B-type natriuretic peptide
BP	Blood pressure
CKD	Chronic kidney disease
CVO	Cardiovascular outcomes
EF	Ejection fraction
HF	Heart failure
HFimpEF	Heart failure with improved ejection fraction
HFmrEF	Heart failure with mildly reduced ejection fraction
HFpEF	Heart failure with preserved ejection fraction
HFrEF	Heart failure with reduced ejection fraction
HR	Hazard ratio
MRA(s)	Mineralocorticoid receptor antagonist(s)
MR	Mineralocorticoid receptor
NYHA	New York Heart Association
SBP	Systolic blood pressure
SGLT2-i	Sodium-glucose co-transporter 2 inhibitor
T2DM	Type 2 diabetes mellitus
TEAE	Treatment-emergent adverse effects
